# Definition and Determination of the Correct Tibial Entry Portal With Accuracy in Intramedullary Nailing of Tibial Fractures

**DOI:** 10.7759/cureus.103650

**Published:** 2026-02-15

**Authors:** Rishabh Saxena, Ashwani Mathur, Nakul Fauzdar, Neel Agarwal, Shataayu Gugale, Shreyansh Gupta, Manasvi Bagree

**Affiliations:** 1 Orthopaedic Surgery, Mahatma Gandhi Medical College and Research Institute, Jaipur, IND

**Keywords:** intramedullary nailing, malalignment, tibial entry point, tibial shaft fracture, trauma

## Abstract

Background

Tibial shaft fractures, frequently caused by high-energy trauma, significantly challenge orthopedic trauma management. Intramedullary interlocking nailing, favored for its biomechanical advantages and reduced complications, critically depends on accurately identifying the tibial entry portal to ensure optimal alignment and functional outcomes.

Methodology

A prospective observational study was designed to evaluate the optimal tibial entry portal for improving alignment outcomes in patients undergoing intramedullary nailing (IMN) for tibial shaft fractures. Conducted at Mahatma Gandhi Medical College and Hospital, Jaipur, the study included 32 adult patients with tibial fractures suitable for IMN. Radiological outcomes and alignment accuracy were assessed at nine-month follow-ups.

Results

Participants had a mean age of 44.63±18.17 years; men predominated (18, 56.3%). The primary injury cause was road traffic accidents (18, 56.3%) and surpassed falls (14, 43.8%) and fracture types were predominantly closed (20, 62.5%), with notable representation of Arbeitsgemeinschaft für Osteosynthesefragen (AO) types 42 A (11, 34.4%) and 42 C (10, 31.3%). Surgical approaches were evenly distributed (16, 50% suprapatellar; 16, 50% infrapatellar). Coronal and sagittal plane malalignments were prevalent, with significant variations linked to entry portal positioning. Complication rates remained low, with delayed union and infection each observed in only one (3.1%) case.

Conclusion

Critical factors influence tibial alignment post-surgery, emphasizing precise entry point selection to reduce malalignment complications and improve patient outcomes.

## Introduction

Tibial shaft fractures are prevalent orthopedic injuries, particularly due to high-energy trauma such as road traffic accidents [1]. The standard treatment is reamed intramedullary interlocking nailing, which allows for biological fixation and early mobilization. However, the success of this procedure heavily depends on accurate placement of the tibial entry point, which influences post-operative alignment and functional outcomes [2,3]. Malalignment can result from improper entry point placement, particularly due to the tibia’s unique anatomy and muscle forces acting on fractured segments. Despite technological advancements, reliance on anatomical landmarks and fluoroscopy for entry point determination introduces variability, leading to angular deformities and inconsistent outcomes [4]. Studies show that misalignment of more than five degrees adversely affects function and increases joint degeneration risk. Yet, literature remains inconsistent regarding the ideal entry point, and most studies are retrospective or based on cadaver models. This study aims to prospectively investigate the correlation between tibial entry point and post-operative alignment in cases managed with reamed intramedullary nailing.

## Materials and methods

A prospective observational clinical study was conducted in the Department of Orthopaedics, Mahatma Gandhi Medical College and Hospital, Jaipur, over a period of 18 months. Ethical approval was obtained from the Institutional Ethics Committee of the institution (approval number: IEC/1475). The study adhered to the principles of the Indian Council of Medical Research (ICMR) Guidelines (2017) and the Declaration of Helsinki (2004). Written informed consent was taken from all participants.

Study population 

All patients admitted with tibial shaft fractures during the study period were screened. Eligible participants were recruited using systematic random sampling.

Inclusion Criteria

The study included adult patients aged 18 years and above presenting with tibial shaft fractures deemed appropriate for intramedullary nailing. Both closed fractures and open fractures classified as Gustilo-Anderson Type I and Type II were eligible for participation.

Exclusion Criteria

Patients were excluded if they had any previous history of tibial shaft fracture or presented with open fractures of Gustilo-Anderson Type III or higher grades. Individuals with a narrow medullary canal, significant tibial deformity, or any radiological or clinical evidence of active joint sepsis were also excluded due to the potential of compromising surgical access, fixation accuracy, or postoperative outcomes.

Data collection

Data were recorded on a semi-structured proforma. Demographic and clinical details included age, sex, occupation, mode of injury, fracture type, associated injuries, and time to intervention. Socioeconomic status was assessed using the modified BG Prasad classification (2022) [5].

Clinical evaluation involved detailed history, examination, and fracture classification (Arbeitsgemeinschaft für Osteosynthesefragen (AO) and Gustilo-Anderson). Radiological assessment comprised anteroposterior and lateral radiographs for pre- and post-operative alignment [6,7].

The Lower Extremity Functional Scale (LEFS) evaluates the functional limitations in lower extremity conditions [8]. The Lysholm Knee Scoring Scale, originally developed by Lysholm and Gillquist and later refined by Tegner and Lysholm [9], was used. 

Data management and confidentiality

Data were entered into Microsoft Excel (Microsoft, Redmond, WA) and analyzed using SPSS version 21.0 (IBM Corp, Armonk, NY). Confidentiality was maintained through anonymization and password-protected data storage.

Statistical analysis

Continuous variables were expressed as mean±standard deviation (SD). Categorical variables as frequency and percentage. Chi-square test (χ²) applied for categorical normal distributed data. Student’s t-test used for continuous variable and p<0.05 was considered statistically significant.

## Results

This study analyzed 32 patients with tibial shaft fractures, focusing on demographic variables, mechanism of injury, fracture types, classifications, malalignment patterns, surgical approaches, postoperative complications, and functional outcomes.

The age of the patients ranged from 20 to 76 years, with a mean age of 44.63 years. The most common age group was those younger than 30 years (31.2%), followed by those older than 60 years (28.1%). Gender distribution showed a male predominance with 18 patients (56.3%) compared to 14 women (43.8%). The most frequent cause of injury was road traffic accidents (56.3%), while falls from height accounted for 43.8% of cases. Closed fractures were the majority (62.5%). Among open fractures, type II was most common (18.8%), followed by type III-A (12.5%). According to the Arbeitsgemeinschaft für Osteosynthesefragen/Orthopaedic Trauma Association (AO/OTA) classification system, type 42 A fractures were the most frequent (34.4%), followed by 42 C (31.3%) and 42 B (28.1%) (Table 1).

**Table 1:** Demographic and Clinical Characteristics of Participants AO: Arbeitsgemeinschaft für Osteosynthesefragen.

Variables	Frequency (n=32)	Percentage (%)
Age Group		
<30 years	10	31.2%
30-45 years	6	18.8%
46-60 years	7	21.9%
>60 years	9	28.1%
Gender		
Male	18	56.3%
Female	14	43.8%
Mode of Injury		
Road traffic accident	18	56.3%
Fall from height	14	43.8%
Fracture Type		
Closed	20	62.5%
Open Type I	1	3.1%
Open Type II	6	18.8%
Open Type III-A	4	12.5%
Open Type III-B	1	3.1%
AO Classification		
42 A	11	34.4%
42 B	9	28.1%
42 C	10	31.3%
Others	2	6.2%

Malalignment distribution

Postoperative radiographs demonstrated coronal plane malalignment most frequently at grade 2 (8, 25.0%), while sagittal plane malalignment was most commonly grade 3 (8, 25.0%). Overall, 11 (34.4%) of patients showed grade ≥3 malalignment in coronal plane and 14 (43.8%) in sagittal plane, indicating clinically significant deformity (Table 2).

**Table 2:** Distribution of Malalignment in Coronal and Sagittal Planes

Grade	Coronal Plane	Sagittal Plane
0	6 (18.8%)	5 (15.6%)
1	7 (21.9%)	7 (21.9%)
2	8 (25.0%)	6 (18.8%)
3	5 (15.6%)	8 (25.0%)
4	6 (18.8%)	6 (18.8%)
Total	32 (100.0%)	32 (100.0%)

**Figure 1:**
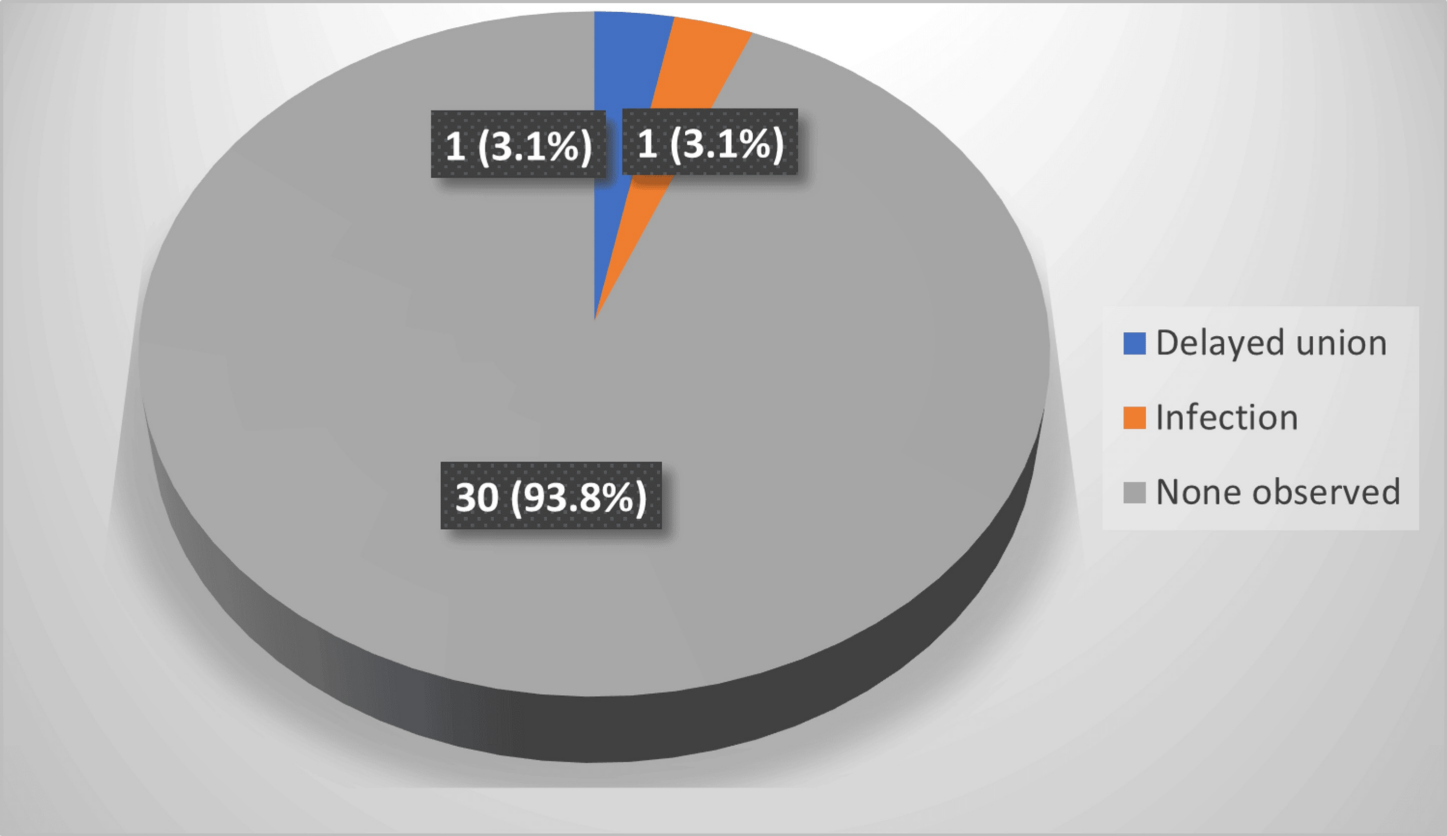
Post-operative Complications

Figure 1 shows that postoperative complications were minimal: 30 (93.8%) patients had uneventful recovery, while one case each of delayed union and infection was reported (1, 3.1% each). Surgical approaches were equally distributed, with 16 (50%) patients undergoing the suprapatellar approach and 16 (50%) patients treated with the infrapatellar approach.

Functional outcomes and union

Table 3 shows functional outcomes were favourable when the mean postoperative knee range of motion was 127.03°±8.79. The Lysholm Knee Scoring Scale yielded a mean of 90.41±7.75, indicating good knee function, while the LEFS averaged 69.63±9.49, reflecting satisfactory lower limb function. The mean time to radiological union was 4.84±1.22 months.

**Table 3:** Baseline Characteristics and Functional Assessment Parameters of Study Participants

Variable	N	Minimum	Maximum	Mean ± SD
Knee range of motion (ROM)	32	110	145	127.03±8.786
Lysholm Knee Scoring Scale	32	63	100	90.41±7.754
Lower Extremity Functional Scale (LEFS)	32	36	80	69.63±9.486
Time of union	32	4	9	4.84±1.221

## Discussion

Our findings align with existing literature indicating higher male predominance and a bimodal age distribution in tibial fractures managed via intramedullary nailing (IMN). The substantial incidence of malalignment emphasizes the critical role of entry portal selection [10]. Comparative analysis revealed the merits of both approaches, highlighting that precision in entry-point localization significantly impacts final alignment outcomes [11,12]. Also, previous cadaveric and clinical studies support thorough preoperative and intraoperative planning [13,14].

A series of studies has highlighted the clinical utility and outcomes of intramedullary interlocking nailing in tibial fractures. Favorable functional recovery has been documented in extra-articular distal tibia fractures treated with tibial interlocking nails [15], and good results have also been reported with dynamic interlock nailing in distal fractures [16]. Reamed interlocking nails have proven effective in managing diaphyseal fractures and aseptic nonunions.

The study comprised 32 patients, with a male predominance (56.3%) similar to findings by Liu et al. (2020) [17]. The mean age was 44.63 years, reflecting a bimodal distribution, suggesting the procedure’s applicability across a wide age spectrum. Similar age-related trends were observed in study by Yadav et al. (2025) [18], though others like Khalil et al. (2020) [19] reported younger cohorts.

Road-traffic accidents (56.3%) were the most common mechanism of injury, followed closely by falls from height (43.8%). The side of the fracture was nearly equally distributed, with a slight right-sided dominance. Most fractures were closed (62.5%), in alignment with findings by Liu et al. (2020) [17]. AO classification showed a balanced distribution among simple, wedge, and complex fractures, with a slight predominance of Type 42A.

Both suprapatellar and infrapatellar approaches were equally employed. While traditional studies favored infrapatellar access, recent evidence, including that from Yadav et al. (2025) [18], supports the rising preference for suprapatellar techniques due to improved alignment outcomes.

The correct tibial entry point continues to be debated, with anatomical localization emphasized in earlier literature [18] and safe surgical techniques elaborated upon in subsequent reports [19]. Technique-related refinements, such as suprapatellar and proximal tibia entry methods, have been further explored, underscoring the importance of fracture level and technical variation [20,21]. Complications remain relevant, with tibial malrotation [22] and anterior knee pain associated with radiological factors [22] being significant concerns. Additionally, the role of fibular integrity in facilitating fracture healing has been established [23], while the efficacy of intramedullary nails as primary fixation in compound tibial fractures has also been demonstrated [24,25]. 

Radiological alignment outcomes were excellent, with 100% achieving acceptable coronal alignment and no sagittal malalignment >5°. The complication rate was low (6.2%), with only isolated cases of delayed union and infection. Functional outcomes, as assessed by Lysholm and LEFS scores, indicated favorable recovery, corroborated by comparable studies. The average time to union was approximately 4.84 months, aligning with existing literature reported by Gadegone et al. (2015) [25] and Patel et al. (2024) [21].

Limitations

The small sample size (n=32) limit the generalizability of findings. Radiographic alignment was measured using plain radiographs; more advanced imaging techniques like CT scans could have provided greater precision in detecting malrotation. This being a single-centric study in a tertiary-care center limits the generalizability of the findings to a broader population.

## Conclusions

Accurate selection and localization of the tibial entry portal play a decisive role in achieving optimal alignment during intramedullary nailing of tibial fractures. Intramedullary nailing remains a reliable and effective method for managing tibial shaft fractures, yielding good radiological and functional outcomes with low complication rates.
